# Isobavachalcone sensitizes cells to E2‐induced paclitaxel resistance by down‐regulating CD44 expression in ER+ breast cancer cells

**DOI:** 10.1111/jcmm.13719

**Published:** 2018-09-04

**Authors:** Junfeng Shi, Yi Chen, Wenxing Chen, Cuiju Tang, Honghong Zhang, Yuetong Chen, Xiuwei Yang, Zhi Xu, Jingsun Wei, Jinfei Chen

**Affiliations:** ^1^ Department of Oncology Nanjing First Hospital NanJing Medical University Nanjing China; ^2^ Clinical Research Center Xuyi People's Hospital Xuyi China; ^3^ Department of Oncology Nanjing Pukou Central Hospital Nanjing China; ^4^ School of pharmacy Nanjing University of Chinese Medicine Nanjing China; ^5^ Department of Pharmacology and Nutritional Sciences University of Kentucky Lexington KY USA; ^6^ Jiangsu Key Lab of Cancer Biomarkers, Prevention and Treatment Collaborative Innovation Center for Cancer Personalized Medicine Nanjing Medical University Nanjing China

**Keywords:** breast cancer, CD44, isobavachalcone, oestrogen receptor alpha, paclitaxel resistance

## Abstract

Oestrogen receptor (ER) is expressed in approximately 60%‐70% of human breast cancer. Clinical trials and retrospective analyses have shown that ER‐positive (ER+) tumours are more tolerant to chemotherapeutic drug resistance than ER‐negative (ER−) tumours. In addition, isobavachalcone (IBC) is known as a kind of phytoestrogen with antitumour effect. However, the underlying mechanism of IBC in ER+ breast cancer needs to be elucidated further. Our in vitro experiments showed that IBC could attenuate 17β‐estradiol (E_2_)‐induced paclitaxel resistance and that E_2_ could stimulate CD44 expression in ER+ breast cancer cells but not in ER− cells. Meanwhile, E_2_ could promote ERα expression to render ER+ breast cancer cells resistant to paclitaxel. Furthermore, we established paclitaxel‐resistant breast cancer cell lines and determined the function of ERα in the enhancement of paclitaxel resistance via the regulation of CD44 transcription. IBC down‐regulated ERα and CD44 expression and thus inhibited tumour growth in paclitaxel‐resistant xenograft models. Overall, our data demonstrated for the first time that IBC could decrease CD44 expression level via the ERα pathway and make ER+ breast cancer cells sensitive to paclitaxel treatment.

## 1 INTRODUCTION

Breast cancer is one of the most common malignant tumours in women, with high rate of morbidity and metastasis.^1^ Oestrogen receptor alpha (ERα) is positive in approximately 60%‐70% of breast cancer patients.^2^, ^3^ Cumulative data show that ERα+ breast cancer cells are more resistant to paclitaxel, a first‐line chemotherapeutic drug for breast cancer, than are ER‐ breast cancer.^4^, ^5^ Therefore, chemotherapy failure caused by paclitaxel resistance is considered a substantial obstacle in the treatment of ER+ breast cancer.^6^, ^7^ However, the underlying mechanism of chemotherapeutic drug resistance in ER+ breast cancer has not yet been elucidated.

17β‐Estradiol (E_2_) can regulate and maintain a series of physiological processes, such as reproduction, CNS development and metabolic balance.^8^ Many cellular responses to E_2_ are mediated by oestrogen receptors (ERs), including ERα and ERβ. Although clinical and experimental data have confirmed the key role of ERα in breast cancer, the role of ERβ in breast cancer remains controversial.^8^, ^9^ Recent studies have demonstrated that expression of ERα can prevent paclitaxel‐induced apoptosis in breast cancer MCF‐7 cells.^10^, ^11^ ERα can act as a nuclear receptor and regulate target gene transcription via the “classical” model or the “nonclassical” model.^12^ In addition to its role in the “classical” model of gene regulation through oestrogen response elements (EREs) in promoter regions, ERα can form protein complexes with other transcription factors, such as Sp1, Ap1 and NF‐κB, to regulate gene transcription involved in the “nonclassical” model.^12^, ^13^, ^14^ These observations suggest that ERα can regulate a large variety of genes that are associated with the development of chemotherapy resistance in ER+ breast cancer cells.

Psoralen, a traditional Chinese herb, is isolated from dried ripe fruits of leguminous *Psoralea corylifolia* L.^15^, ^16^, ^17^ It is warm natured and pungent flavoured, with the effect of enriching the kidney and strengthening yang.^18^ Recent studies have shown that psoralen has some biological functions, such as blood vessel dilatation, myocardial contractility enhancement and antifungal, anticancer and oestrogen‐like effects.^19^ Modern pharmacological studies have also shown that isobavachalcone (IBC), an important component of psoralen, has strong antibacterial, antioxidant, anti‐reverse transcriptase, antitubercular and anticancer abilities.^20^, ^21^ Previous studies have reported that IBC inhibits tumour formation in mouse skin cancer and induces apoptosis in neuroblastoma.^22^, ^23^ However, the potential functions of IBC in cancer‐related treatment need further study.

CD44 and CD24 are characteristic of the cancer stem cell phenotype, and these molecules are closely associated with poor prognosis and chemotherapy resistance in cancer.^24^, ^25^, ^26^, ^27^ Recently, natural substances from plants have been documented as effective intervention agents in the down‐regulation of CD44/CD24 expression in experimental breast carcinoma.^28^ However, whether IBC can directly regulate CD44/CD24 expression to decrease paclitaxel resistance in ER+ breast cancer cells remains unclear. This study aimed to explore whether IBC influences resistance of breast cancer cells to paclitaxel by regulating CD44/CD24 expression. In this study, first, we aimed to establish a close correlation between CD44 and ERα expression in ER+ breast cancer cells with oestrogen stimulation or the development of paclitaxel resistance. Second, we explored the function of ERα in the enhancement of paclitaxel resistance via the regulation of CD44 expression. Finally, we determined that IBC could enhance the sensitivity of paclitaxel‐resistant breast cancer cells and reduce the growth of xenograft tumours via the regulation of CD44 expression. Taken together, for the first time, our results demonstrated that inhibition of ERα by IBC can down‐regulate CD44 expression and thus decrease paclitaxel resistance in ER+ breast cancer cells and xenograft tumour models.

## 2 MATERIALS AND METHODS

### 2.1 Cell culture and chemicals

The human breast cancer cell lines ZR‐75‐1, MCF‐7 and MDA‐MB‐231 were obtained from the ATCC. ZR‐75‐1 cells and ZR‐75‐1/R cells were cultured in DMEM; MCF‐7 cells and MCF‐7/R cells were cultured in EMEM; and MDA‐MB‐231 cells were cultured in L‐15 medium. All culture media, containing 10% (v/v) foetal bovine serum, penicillin (200 U/mL) and streptomycin (100 μg/mL), were purchased from Gibco Life Technology (Grand Island, NY, USA). Paclitaxel (Taxol), E_2_, IBC and 3‐(4,5‐dimethylthiazol‐2‐yl)‐2,5‐diphenyltetrazolium bromide (MTT) were obtained from Sigma (St. Louis, MO, USA). Antibodies against ERα and P‐gp were purchased from Abcam (Cambridge, MA, USA). The anti‐CD44 antibody was purchased from Proteintech (Proteintech Group, Chicago, IL, USA).

### 2.2 Stepwise selection of cells

We simulated the development of resistance in clinics by weekly treating ZR‐75‐1 and MCF‐7 cells with paclitaxel to generate paclitaxel‐resistant cell lines. ZR‐75‐1 and MCF‐7 cells were treated in a stepwise manner with increasing concentrations of paclitaxel (beginning concentration at 2.5 nmol/L and final concentration at 50 nmol/L) to generate ZR‐75‐1/R and MCF‐7/R cells after 8 months. The resistance index (RI) of cell variants represents the IC_50_ value of paclitaxel‐resistant ZR‐75‐1/R and MCF‐7/R cells divided by the IC_50_ value of the parental ZR‐75‐1 and MCF‐7 cells for each dose of paclitaxel tested.

### 2.3 Cell viability assay

ZR‐75‐1 and MCF‐7 cells were seeded at 5000 cells per well in 96‐well plates and then treated with the indicated concentrations of paclitaxel (72 hours) or E_2_/IBC (48 hours). Subsequently, the cells were treated with 10 μL MTT (5 mg/mL) at 37°C for 4 hours followed by 150 μL dimethyl sulphoxide, and cell viability was determined by measuring the absorbance at 570 nm using a microplate reader (Bio‐Rad, California, USA).

### 2.4 RNA isolation and real‐time PCR

Total RNA was isolated using TRIzol reagent (Invitrogen) according to the manufacturer's instructions. Approximately 1 μg of extracted RNA was reverse transcribed to cDNA using random primers. Real‐time PCR was performed with cDNA using SYBR green (TOYOBO). The primers used were as follows: CD44 (forward 5′‐CGCTATGTCCAGAAAGGAGAAT‐3′ and reverse 5′‐CTGCTCACGTCATCATCAGTAG‐3′); CD24 (forward 5′‐TCAAGTAACTCCTCCCAGAGTA‐3′ and reverse 5′‐AGAGAGTGAGACCACGAAGA‐3′); and GAPDH (forward 5′‐CAGGGCTGCTTTTAACTCTGGTAA‐3′ and reverse 5′‐GGGTGGAATCATATTGGAACATGT‐3′).

### 2.5 Transient transfection

Cells were seeded and transfected with Lipofectamine^TM^ 2000 (Invitrogen, Shanghai, China) according to the manufacturer's protocol. ZR‐75‐1, MCF‐7, ZR‐75‐1/R and MCF‐7/R cells were plated in six‐well plates at a density of 1 × 10^6^ cells/well. The cells were transfected with pcDNA3.1‐ERα (ERα expression vector), pRNAT‐H1.1‐ERα‐shRNA (ERα‐shRNA) and si‐CD44 (CD44‐targeting siRNA) plasmids using Lipofectamine^TM^ 2000. ERα‐targeted shRNA was constructed and described in our previously work.^29^ The siRNA used for silencing CD44 gene was purchased from GenePharma (Shanghai, China). The sequences for siCD44‐1 and siCD44‐2 were as follows: sense 5′ GGACCAAUUACCAUAACUATT 3′, antisense 5′ UAGUUAUGGUAAUUGGUCCTT 3′ and sense 5′ GCAGUCAACAGUCGAAGAATT 3′, antisense 5′ UUCUUCGACUGUUGACUGCTT 3′. As a negative control, pcDNA3.1, pGC‐control‐shRNA and si‐control were used.

### 2.6 Western blotting

Total protein was extracted from cultured cells, subjected to 8%‐12% SDS‐polyacrylamide gel electrophoresis and transferred onto 0.45‐μm PVDF membranes (Millipore). The membranes were then blocked with 5% milk‐TBST for 1 hour and incubated with primary antibodies against CD44 (diluted 1:1000), ERα (diluted 1:1000), P‐gp (diluted 1:1000) and β‐actin (diluted 1:5000 in TBST) overnight. After incubation with appropriate secondary antibodies for 1 hour at room temperature, the membranes were detected by Tanon 5200 Imaging System (Shanghai, China).

### 2.7 Clonogenic cell survival assay

Cells suspended in fresh culture medium were plated at a density of 400 cells/well onto 6‐cm plates and incubated for 24 hours and then cultured in fresh medium containing 5 nmol/L paclitaxel. At the end of 2 weeks of incubation, the cells were fixed with cold methanol and stained with crystal violet (Beyotime, China) for 20 minutes at room temperature. Thereafter, the plates were gently washed with water and allowed to air‐dry. All experiments were performed in triplicate.

### 2.8 Flow cytometry assay

Approximately 10^6^ cultured cells were harvested and fixed in 75% ethanol diluted in PBS at 20°C overnight. The cells were then incubated in PBS containing 100 μg/mL propidium iodide (PI), 100 μg/mL RNase and 0.1% Triton X‐100 at room temperature for 30 minutes before flow cytometry analysis. Cell cycle distribution and DNA content were determined using a BD FACSCalibur system (Becton, Dickinson and Company, USA).

### 2.9 Tumour xenograft and immunohistochemistry assay

Animal experiments were approved by the Animal Care Committee of Nanjing First Hospital, Nanjing Medical University (Approval No. SYXK20160006). Approximately 5 × 10^6^ MCF‐7/R cells were injected into the mammary fat pads of 5‐week‐old female athymic nude mice. The mice were randomly assigned into two groups of six. When the size of the xenograft reached approximately 50 mm^3^, IBC was intraperitoneally administered daily at 100 mg/kg into the nude mice. Tumour volumes were calculated at the indicated time‐points with the formula: π/6 × length × width2. Tumour samples from the mice were deparaffinized and rehydrated. After blocking the endogenous peroxidase activity, the sections incubated with primary monoclonal anti‐ERα and anti‐CD44 antibodies at a dilution of 1:500 overnight at 4°C. The sections were incubated with appropriate HRP‐conjugated secondary antibody for 1 hour at room temperature. Colour was developed with 3‐amino‐9‐ethylcarbazole solution.

Image pro plus (IPP) was used as the method of quantitative image analysis on immunohistochemical staining, evaluating the expressing protein of ERα and CD44 gene by IBC treatment. Firstly, the positive area (ERα or CD44 protein staining) in the image was taken as AOI (area of interest). Then the value of SA (sum area) and IOD (integrated optical density) was measured by IPP software, and the value of MOD (mean optical density) was calculated as the following formula: MOD = IOD/SA.

### 2.10 Statistical analysis

Each of the experiments was repeated at least three times. Values are expressed as the mean ± SD of triplicate measurements unless otherwise noted. Student's paired *t* test was used to analyse differences between the sample of interest and its control. *P* < .05 was considered statistically significant.

## 3 ReSults

### 3.1 IBC decreased resistance of ER+ breast cancer to paclitaxel

**Figure 1:**
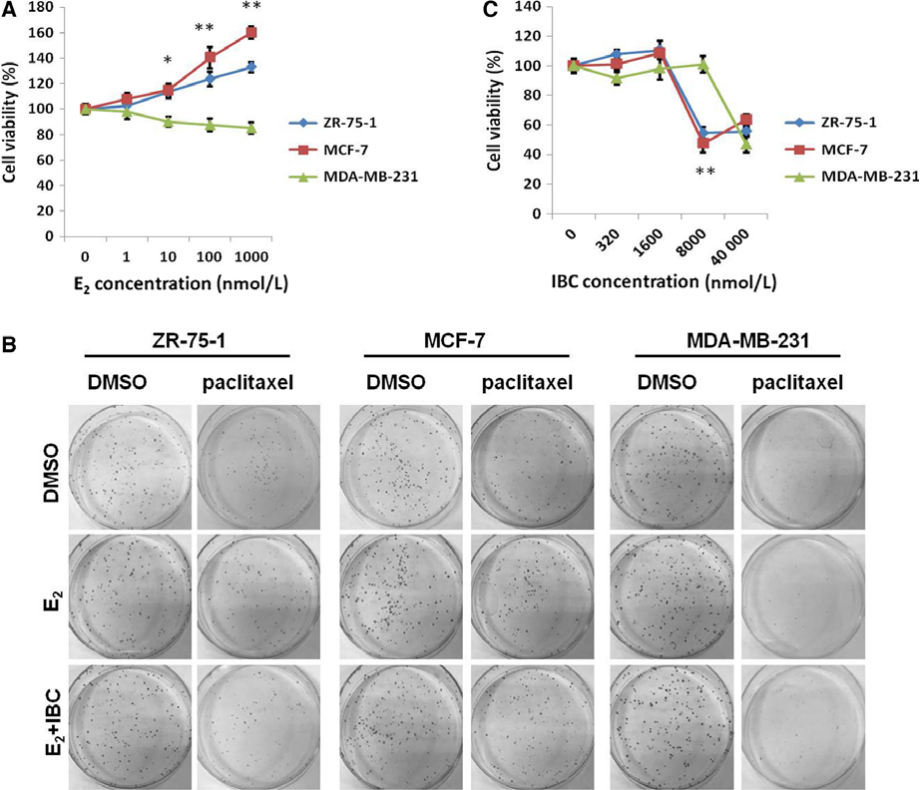
IBC decreased paclitaxel resistance in ER+ breast cancer. A, ZR‐75‐1 (ER+), MCF‐7 (ER+) and MDA‐MB‐231 (ER−) breast cancer cells were treated with the indicated concentrations of E_2_ for 72 h and subjected to cell viability assay. B, ER+ and ER− breast cancer cells were treated with E_2_ (1 μmol/L) for 24 h and subsequently treated with the indicated concentrations of IBC for 72 h. C, ER+ and ER− breast cancer cells were pretreated with E_2_ (1 μmol/L) or its combination with IBC (8 μmol/L) for 72 h and subsequently treated with paclitaxel (5 nmol/L) for 14 d before being subjected to colony formation assay. *P < .05, **P < .01

Although oestrogen can maintain biological functions in breast cancer cells, its roles in the stimulation of drug resistance in breast cancer cells are still controversial. Therefore, we analysed the viable number of breast cancer cells after treatment with different concentrations of E_2_. Our data indicated that the number of viable cells displayed an increasing tendency in ER+ ZR‐75‐1 and MCF‐7 cells but not in ER‐ MDA‐MB‐231 cells (Figure 1A). After 2 weeks of incubation with 5 nmol/L paclitaxel, the number of colonies formed by ZR‐75‐1 and MCF‐7 cells in the E_2_ group was higher than that in the control group (Figure 1B), suggesting that E_2_ could promote the proliferation of ER+ breast cancer cells to make them resistant to paclitaxel.

IBC is well‐known as a kind of phytoestrogen, but its therapeutic mechanism in breast cancer remains unclear. To investigate the effect of IBC on breast cancer cells, we carried out cell viability assays in the presence of 1 μmol/L E_2_. Our results demonstrated that the viability significantly decreased in ZR‐75‐1 and MCF‐7 cells treated with 8 μmol/L IBC (Figure 1C). In addition, fewer colonies of ZR‐75‐1 and MCF‐7 cells were formed in the E_2_+IBC group than in the E_2_ group (Figure 1B). These findings indicated that E_2_ could increase the resistance of ER+ breast cancer cells to paclitaxel, while IBC could attenuate E_2_‐induced paclitaxel resistance.

### 3.2 CD44 was up‐regulated by E_2_ in ER+ breast cancer cells

**Figure 2:**
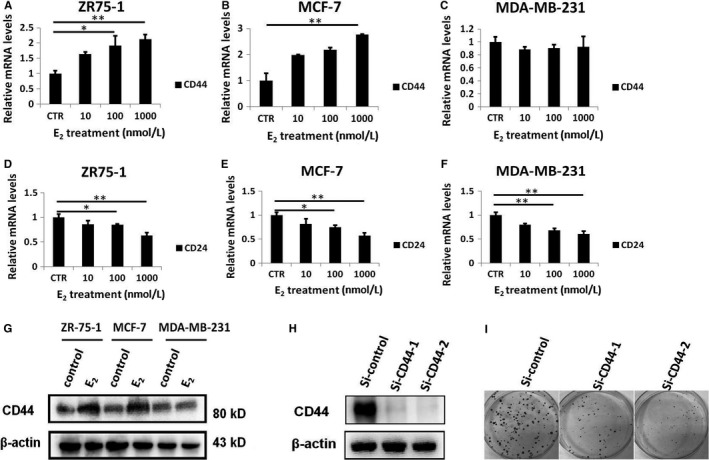
E_2_ could stimulate CD44 expression in ER+ breast cancer cells. A, CD44 transcript level was detected by real‐time PCR in ER+ ZR‐75‐1 cells treated with the indicated concentrations of E_2_ for 72 h. B, CD44 transcript level was detected by real‐time PCR in ER+ MCF‐7 cells treated with the indicated concentrations of E_2_ for 72 h. C, CD44 transcript level was detected by real‐time PCR in ER‐ MDA‐MB‐231 cells treated with the indicated concentrations of E_2_ for 72 h. D, Real‐time PCR analysis of CD24 expression in ER+ ZR‐75‐1 cells treated with the indicated concentrations of E_2_ for 72 h. E, Real‐time PCR analysis of CD24 expression in ER+ MCF‐7 cells treated with the indicated concentrations of E_2_ for 72 h. F, Real‐time PCR analysis of CD24 expression in ER+ MCF‐7 cells treated with the indicated concentrations of E_2_ for 72 h. G, Western blot analysis of CD44 protein expression in breast cancer cells treated with E_2_ (1 μmol/L) for 72 h. H, Western blot analysis of CD44 protein expression in ER+ MCF‐7 cells transfected with CD44‐targeting siRNA. H, Western blot analysis of CD44 protein expression in ER+ MCF‐7 cells transfected with CD44‐targeting siRNA. I, MCF‐7 cells described in Figure 1 were treated with paclitaxel (5 nmol/L) for 14 d before being subjected to colony formation assay. *P < .05, **P < .01

Although CD44 and CD24 expression is a poor prognostic marker for different tumours including breast cancer,^24^, ^30^ the role of CD44 and CD24 in paclitaxel resistance of breast cancer cells stimulated by E_2_ needs further study. Therefore, we analysed CD44 and CD24 expression in breast cancer cells treated with different concentrations of E_2_ using real‐time PCR. Indeed, in comparison with that in ER‐ MDA‐MB‐231 cells (Figure 2C), the expression of CD44 displayed an increasing tendency with increasing E_2_ concentration in ER+ ZR‐75‐1 and MCF‐7 cells (Figure 2A and B). In addition, the expression levels of CD24 mRNA decreased in all breast cancer cells after stimulation with the same concentration of E_2_ (Figure 2D, E and F). Western blotting results further demonstrated that the expression levels of the CD44 protein were specifically elevated in ZR‐75‐1 and MCF‐7 cells after E_2_ stimulation (Figure 2G). These results indicated that CD44 plays an important role in ER+ breast cancer cells in the presence of E_2_ stimulation. To further identify the function of CD44 during the response to paclitaxel, we performed siRNA‐mediated knockdown of CD44 in MCF‐7 cells (Figure 2H). Colony formation assay showed that fewer paclitaxel‐resistant colonies were formed in the si‐CD44 group, which was, than in the control group (Figure 2I). These data suggested that CD44 expression might be involved in the resistance to paclitaxel‐mediated E_2_ stimulation.

### 3.3 ERα induced by E_2_ enhanced CD44 expression in breast cancer cells

**Figure 3:**
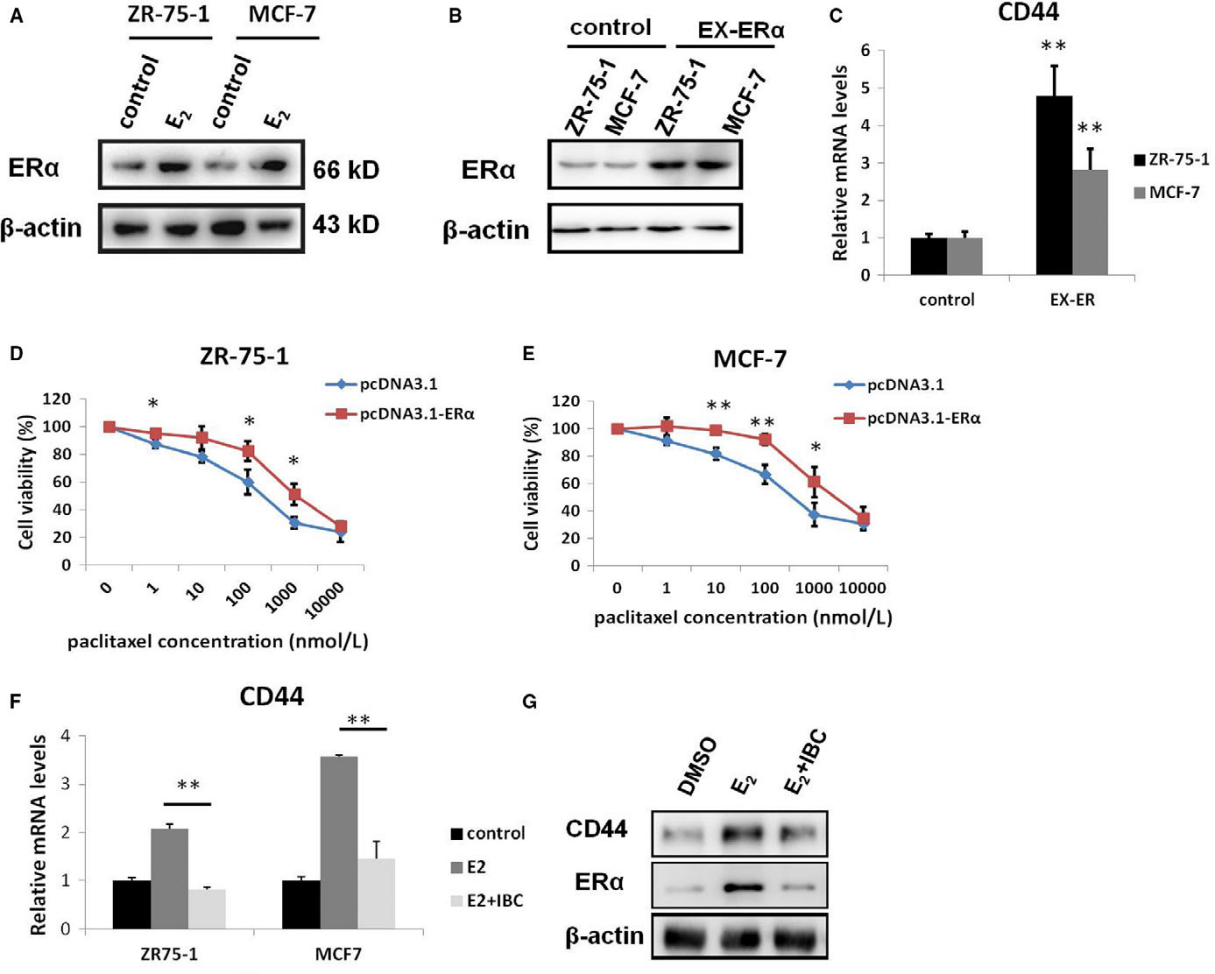
ERα could enhance CD44 expression to promote the resistance of ER+ breast cancer cells to paclitaxel. A, Western blot analysis of ERα protein expression in ER+ breast cancer cells treated with E_2_ (1 μmol/L). B, Western blot analysis of ERα protein expression in ZR‐75‐1 and MCF‐7 cells transfected with empty vector or ERα expression plasmids. C, Real‐time PCR analysis of CD44 expression in ZR‐75‐1 and MCF‐7 cells transfected with vector or ERα expression plasmids. D, ZR‐75‐1 cells were treated with the indicated concentrations of paclitaxel for 72 h and subjected to cell viability assay. E, MCF‐7 cells were treated with the indicated concentrations of paclitaxel for 72 h and subjected to cell viability assay. F, Real‐time PCR analysis of CD44 transcript expression in both ZR‐75‐1 and MCF‐7 cells treated with E_2_ (100 nmol/L) or its combination with IBC (8 μmol/L) for 72 h. G, Western blot analysis of ERα and CD44 protein expression in MCF‐7 and MCF‐7/R cells treated with E_2_ (1 μmol/L) or its combination with IBC (8 μmol/L) for 72 h. *P < .05, **P < .01

Our previous study showed that ERα was essentially involved in chemoresistance, which may be attributed to its role as a nuclear transcriptional factor in regulating some important tumour drug‐resistant genes.^10^ Thus, we detected the expression of ERα in ER+ breast cancer cells stimulated by E_2_. Western blotting displayed that E_2_ could increase ERα expression levels in ZR‐75‐1 and MCF‐7 cells (Figure 3A), suggesting a significant positive correlation between ERα and CD44 in ER+ breast cancer cells. To further explore whether CD44 expression was regulated by ERα, we examined the expression of CD44 in ZR‐75‐1 and MCF‐7 cells transfected with ERα. The transfection efficiency of the ERα expression vector was confirmed by Western blotting (Figure 3B). Additionally, real‐time PCR showed that compared with that in the cells transfected with control plasmids, the expression of CD44 mRNA was up‐regulated in paclitaxel‐sensitive cells transfected with ERα (Fig. 3C). Moreover, the viability of ZR‐75‐1 and MCF‐7 cells transfected with ERα was higher than that of cells transfected with control plasmids (Figure 3D and E). Interestingly, IBC could clearly reverse the increase in expression of ERα and CD44 stimulated by E_2_ as shown by real‐time PCR and Western blotting (Figure 3F and G). Taken together, these results suggested that IBC could decrease CD44 expression in an ERα‐dependent manner.

### 3.4 Establishment of paclitaxel‐resistant ZR‐75‐1/R and MCF‐7/R cell lines

**Figure 4:**
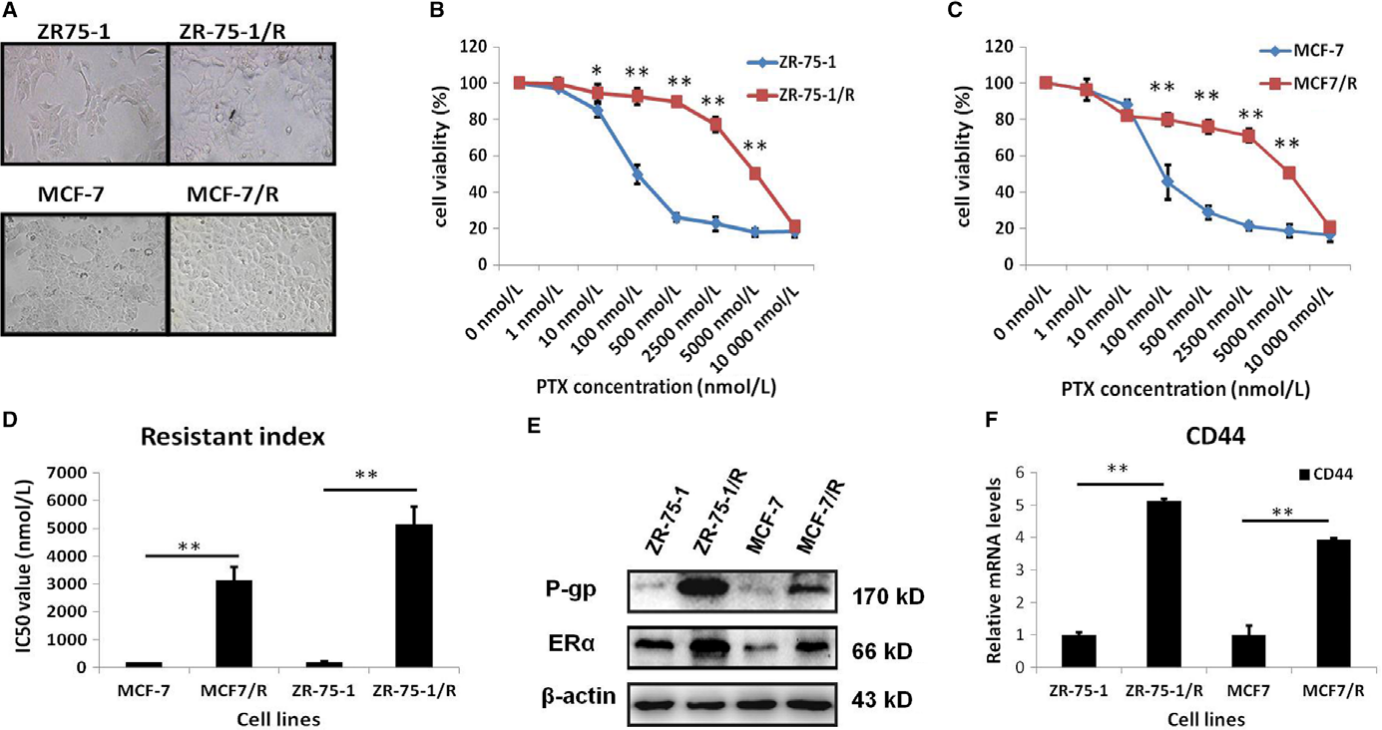
The expression of ERα and CD44 increased simultaneously in paclitaxel‐resistant ER+ breast cancer cells we established. A, Morphology of paclitaxel‐resistant ZR‐75‐1/R and MCF‐7/R cells. B, Number of viable paclitaxel‐resistant ZR‐75‐1/R cells and their parental cells treated with the indicated concentrations of paclitaxel for 72 h as determined by cell viability assay. C, Number of viable paclitaxel‐resistant MCF‐7/R cells and their parental cells treated with the indicated concentrations of paclitaxel for 72 h as determined by cell viability assay. D, IC_50_ values were calculated in paclitaxel‐resistant cells and their parental cells from B and C. E, Western blot analysis of P‐gp and ERα protein expression in paclitaxel‐resistant cells and their parental cells. F, Real‐time PCR analysis of CD44 transcript level in paclitaxel‐resistant cells and their parental cells. *P < .05, **P < .01

To study the roles of ERα and CD44 in the acquisition of resistance to anticancer agents, we selected ERα+ breast cancer cells to establish drug‐resistant cell models. As mentioned in Materials and Methods, paclitaxel‐sensitive ZR‐75‐1 and MCF‐7 cells were first treated with 2.5 nmol/L paclitaxel. When ZR‐75‐1 and MCF‐7 cells survived at any given concentration of the drug, the cells were passaged at another concentration that was 1.5‐ to twofold higher. After stepwise selection with increasing paclitaxel concentrations, we obtained ZR‐75‐1/R and MCF‐7/R cells, which were able to survive in 50 nmol/L paclitaxel (Figure 4A). Then, cytotoxicity assays were carried out in ZR‐75‐1/R and MCF‐7/R cells to characterize resistance phenotypes. The results demonstrated that the viability of paclitaxel‐resistant cells was higher than that of paclitaxel‐sensitive cells (Figure 4B and C). Furthermore, the IC_50_ values of ZR‐75‐1 and ZR‐75‐1/R cells were 191.3 ± 21.78 and 5151.64 ± 631.08, respectively, whereas the IC_50_ values of MCF‐7 and MCF‐7/R cells were 188.71 ± 15.96 and 3147.44 ± 469.1, respectively (Figure 4D). The RI of the established ZR‐75‐1/R and MCF‐7/R cells was 26.9 and 16.8, respectively, and this result sufficiently demonstrated that those two cell lines were resistant to paclitaxel. It is known that high expression of multidrug resistance protein P‐gp makes cancer cells tolerant to chemotherapeutic drugs.^31^, ^32^ Therefore, we detected P‐gp expression in the paclitaxel‐resistant cell models. Compared with that in paclitaxel‐sensitive cells, the expression level of P‐gp was significantly increased in paclitaxel‐resistant cells (Figure 4E), which indicated that ZR‐75‐1/R and MCF‐7/R cells were chemotherapeutic drug‐resistant breast cancer cells. Interestingly, compared with those in paclitaxel‐sensitive cells, the expression levels of ERα and CD44 were also increased in paclitaxel‐resistant cells (Figure 4E and F), suggesting that ERα and CD44 are up‐regulated by paclitaxel and might be involved in the acquisition of the drug resistance phenotype in ERα+ breast cancer cells. This was consistent with our previous findings and strongly suggested that ERα expression was significantly positively correlated with CD44 expression in paclitaxel‐resistant breast cancer cells.

### 3.5 Knockdown of ERα significantly decreased CD44 gene expression to render breast cancer cells sensitive to paclitaxel

**Figure 5:**
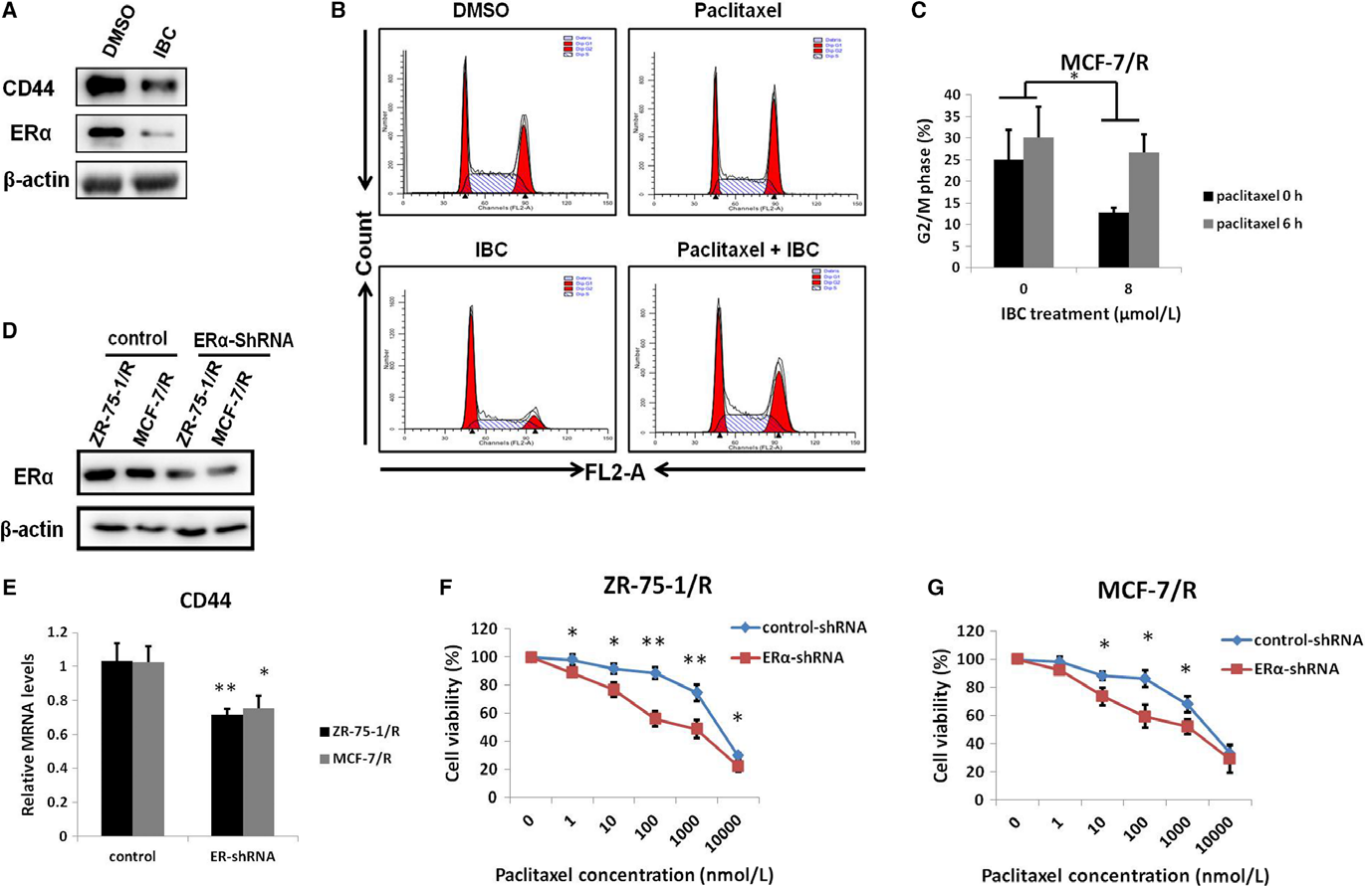
Knockdown of ERα significantly decreased CD44 gene expression to sensitize breast cancer cells to paclitaxel. A, Western blot analysis of ERα and CD44 protein expression in MCF‐7/R cells treated IBC (8 μmol/L) for 72 h. B, MCF‐7/R cells were pretreated with control or IBC (8 μmol/L) for 72 h and subsequently treated with 100 nmol/L paclitaxel for 6 h and subjected to flow cytometry for the analysis of cell cycle distribution. C, Quantitative analysis of the percentage of cells in G2/M phase from three independent experiments is shown. D, Western blot analysis of ERα protein expression in paclitaxel‐resistant ZR‐75‐1/R and MCF‐7/R cells transfected with empty vector or ERα knockdown plasmids. E, Real‐time PCR analysis of CD44 expression in ZR‐75‐1/R and MCF‐7/R cells transfected with empty vector or ERα knockdown plasmids. F, ZR‐75‐1/R cells were treated with the indicated concentrations of paclitaxel for 72 h and subjected to cell viability assay. G, MCF‐7/R cells were treated with the indicated concentrations of paclitaxel for 72 h and subjected to cell viability assay. *P < .05, **P < .01

To evaluate the function of IBC in the paclitaxel resistance phenotypes of ERα+ breast cancer cells, we detected the protein expression of ERα and CD44 in paclitaxel‐resistant breast cancer cells with IBC treatment. Indeed, compared with that in control cells, the expression of ERα and CD44 protein was down‐regulated in paclitaxel‐resistant cells treated with IBC (Figure 5A). Paclitaxel blocks the G2/M phase of cell cycle and induces cancer cell apoptosis.^33^ As shown in Figure 5B and C, IBC could increase the proportion of cells in G2/M phase as determined by flow cytometry in MCF‐7/R cells treated with paclitaxel. These results indicated that IBC sensitized cells to paclitaxel to some extent by blocking cell cycle progression in ER+ breast cancer cells. To further confirm whether IBC reduced the expression of CD44 by regulating the ERα pathway, we carried out the ERα interference experiment in paclitaxel‐resistant breast cancer cells. The efficiency of ERα knockdown by ERα‐shRNA was confirmed by Western blotting (Figure 5D). Real‐time PCR showed that compared with that in cells transfected with control plasmids, the expression of CD44 mRNA was down‐regulated in paclitaxel‐resistant cells transfected with ERα‐shRNA (Figure 5E). In addition, knockdown of ERα expression with ERα‐shRNA significantly decreased the number of viable ZR‐75‐1/R and MCF‐7/R cells (Figure 5F and G), indicating that IBC could down‐regulate CD44 expression by the ERα pathway to sensitize of resistant breast cancer cells to paclitaxel.

### 3.6 IBC antagonized tumour growth by reducing ERα and CD44 expression in a paclitaxel‐resistant mouse xenograft model

**Figure 6:**
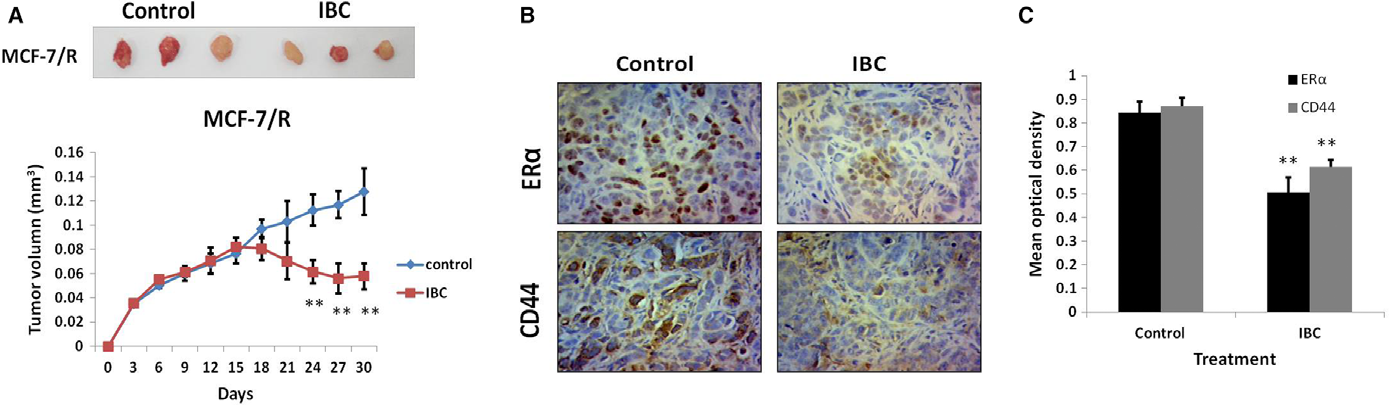
IBC down‐regulated CD44 expression mediated by the ERα pathway and inhibited tumour growth in MCF‐7/R xenograft models. A, MCF‐7/R cells were subcutaneously injected into the right flank of nude mice (6 mice per group) on day 0. When the tumour volume reached 50 mm^3^, the mice were intraperitoneally administered with IBC (100 mg/kg) daily for 24 d. Tumour volume was measured at the indicated time‐points. B, Representative immunohistochemistry images of ERα and CD44 staining in tumour tissues of nude mice. C, Quantitative image analysis utilized IPP software on immunohistochemical staining described in Figure 6B of ERα and CD44 protein expression. *P < .05, **P < .01

To evaluate the clinical implications of our findings, we also studied the functional effect of IBC in vivo in established paclitaxel‐resistant breast cancer xenograft models. Treatment with IBC reduced tumour growth in the mouse xenograft model (Figure 6A). In addition, the levels of ERα and CD44 were lower in the IBC group than in the control group by immunohistochemical (IHC) staining and IPP software analysis (Figure 6B and C), and a positive correlation was observed between ERα and CD44 in MCF‐7/R mouse xenograft tumours treated with IBC. These results suggested that IBC displayed an antitumour activity in MCF‐7/R xenograft tumour models.

## 4 DISCUSSION

Drug resistance is one of the major obstacles limiting the success of cancer chemotherapy. In recent years, both clinical observations and experimental studies have shown that the therapeutic efficacy of anticancer drugs can be altered by steroid hormones and their receptors, but the potential molecular mechanisms remain unclear.^34^, ^35^, ^36^ In this study, using our established paclitaxel‐resistant breast cancer cells, we explored whether and how IBC influenced paclitaxel resistance in breast cancer cells via the ERα‐dependent pathway. First, we verified that IBC can reverse E_2_‐induced paclitaxel resistance in ER+ breast cancer cells. Furthermore, we confirmed a positive correlation between the expression level of ERα and CD44 in our established paclitaxel‐resistant ER+ breast cancer cells. Moreover, knockdown of ERα expression by shRNA or IBC increased the sensitivity of cells with a decreased expression of CD44 to paclitaxel. Finally, using xenograft tumour models, we confirmed that IBC could reduce tumour growth in ER+ xenografts by inhibiting ERα and CD44 expression.

De novo anti‐oestrogen resistance is observed mainly in ER‐/PR‐ tumours, but cancer cells can sometimes switch the phenotype from ER+ to ER−.^37^, ^38^ Based on failure to endocrine therapy, the patients can be treated with chemical therapeutic agents. Paclitaxel, a first‐line clinical chemotherapy drug, is used in the treatment of various human solid tumours, including breast cancer.^39^, ^40^ However, some types of breast tumours are resistant to paclitaxel. Cumulative data from clinical studies and retrospective studies indicate that the ER status might affect the therapeutic efficacy of paclitaxel because paclitaxel is less effective in patients with ER+ breast tumours than in patients with ER‐ breast tumours.^5^, ^41^ However, most of these data were obtained from comparative studies in tumour cell lines extracted from different patients. Thus, it is difficult to reach a consensus on cellular and molecular mechanisms in these cell lines. Therefore, the comparison of established paclitaxel‐resistant breast cell lines and parental breast cell lines may provide us with a valuable model system to explore the mechanism underlying ERα‐mediated resistance to paclitaxel and other chemotherapeutic agents in breast tumour cells.

IBC is a traditional Chinese medicine that has been used as an antibacterial and antitumour agent. Recent studies support the notion that IBC can inhibit human tongue and liver cancer cells by blocking the Akt signalling pathway.^21^ However, we observed no connection between IBC treatment and drug‐resistant breast cancer cells. It is obvious that the relationship between IBC and drug resistance needs to be further elucidated in greater detail. Our results have shown that IBC could down‐regulate the expression of ERα and CD44, leading to cellular sensitivity to paclitaxel. However, it is worth investigating whether IBC can be used to treat other drug‐resistant cancer cells. It is well‐known that E_2_ can promote the proliferation of breast cancer cells. This study indicated that E_2_ could active ERα expression to promote drug resistance in ER+ breast cancer. However, the effect of IBC as a phytoestrogen on ERα is not clear. Our work further demonstrated that IBC could down‐regulate ERα expression to prevent ERα‐related resistance of tumour cells. In this regard, the present study provided a potential antitumour mechanism of IBC.

In this study, for the first time, we correlated ERα and CD44 expression, revealing a potential mechanism that ERα could enhance paclitaxel resistance in ER+ breast cancer cells by up‐regulating CD44 expression. In our study, we proposed the following drug resistance model: during paclitaxel therapy for breast cancer, paclitaxel can active ERα expression and then ERα can transcriptionally regulate the CD44 gene, which is responsible for resistance to chemotherapy agents. Therefore, ERα can be developed as a therapeutic target in the context of CD44 activation to overcome drug resistance in the clinic.

CD44 is closely associated with chemotherapeutic drug resistance in cancer cells. Previous studies have revealed that CD44 is highly expressed in triple‐negative breast cancer (TNBC) and correlated with poor survival of TNBC patients.^42^ Of note, CD44 also plays a key role in ER+ breast cancer, resulting in poor prognosis and radiotherapy resistance.^42^ However, the role of CD44 in drug resistance of ER+ breast cancer is worth studying in depth. Interestingly, in this study, a high expression of CD44 was positively correlated with ERα existed in our established paclitaxel‐resistant ER+ breast cancer cells. It is worth investigating whether the high expression of CD44 increased the resistance of cells. The present study proved that the high expression of CD44 mediated by E_2_‐ERα is an important factor rendering cells resistant to paclitaxel. This is in agreement with the notion that IBC down‐regulates CD44 expression by the ERα pathway to sensitize breast cancer cells to paclitaxel. In conclusion, the present study provided a potential antitumour mechanism of IBC.

In summary, the present study demonstrated that the ER status might play a significant role in determining the sensitivity of breast cancer to paclitaxel. The results obtained from this study may provide valuable information for improving the understanding of CD44‐mediated resistance to paclitaxel and the clinical application of this class of antineoplastic drugs. Further studies are necessary to elucidate the resistance phenotype by which CD44 expression is regulated by ERα in other hormone‐dependent tumours. These studies will help provide a new possibility for the clinical treatment of cancer in premenopausal women.

## CONFLICTS OF INTEREST

The authors confirm that there are no conflicts of interest.

## References

[1] Morikawa A , Wang R , Patil S , et al. Characteristics and prognostic factors for patients with HER2‐overexpressing breast cancer and brain metastases in the era of HER2‐targeted therapy: an argument for earlier detection. Clin Breast Cancer. 2017; Dec 21. pii: S1526‐8209(17)30394‐4. 10.1016/j.clbc.2017.12.009 29337140

[2] Selli C , Dixon JM , Sims AH . Accurate prediction of response to endocrine therapy in breast cancer patients: current and future biomarkers. Breast Cancer Res. 2016;18:118. 27903276 10.1186/s13058-016-0779-0 PMC5131493

[3] Plaza‐Menacho I , Morandi A , Robertson D , et al. Targeting the receptor tyrosine kinase RET sensitizes breast cancer cells to tamoxifen treatment and reveals a role for RET in endocrine resistance. Oncogene. 2010;29:4648‐4657. 20531297 10.1038/onc.2010.209

[4] Tokuda E , Seino Y , Arakawa A , et al. Estrogen receptor‐alpha directly regulates sensitivity to paclitaxel in neoadjuvant chemotherapy for breast cancer. Breast Cancer Res Treat. 2012;133:427‐436. 21909982 10.1007/s10549-011-1758-x

[5] Colleoni M , Bagnardi V , Rotmensz N , et al. Increasing steroid hormone receptors expression defines breast cancer subtypes non responsive to preoperative chemotherapy. Breast Cancer Res Treat. 2009;116:359‐369. 18941889 10.1007/s10549-008-0223-y

[6] Sun S , Liang X , Zhang X , et al. Phosphoglycerate kinase‐1 is a predictor of poor survival and a novel prognostic biomarker of chemoresistance to paclitaxel treatment in breast cancer. Br J Cancer. 2015;112:1332‐1339. 25867275 10.1038/bjc.2015.114 PMC4402453

[7] Ikeda H , Taira N , Nogami T , et al. Combination treatment with fulvestrant and various cytotoxic agents (doxorubicin, paclitaxel, docetaxel, vinorelbine, and 5‐fluorouracil) has a synergistic effect in estrogen receptor‐positive breast cancer. Cancer Sci. 2011;102:2038‐2042. 21801281 10.1111/j.1349-7006.2011.02050.x

[8] Nilsson S , Gustafsson JA . Biological role of estrogen and estrogen receptors. Crit Rev Biochem Mol Biol. 2002;37:1‐28. 11905545 10.1080/10409230290771438

[9] Arpino G , Wiechmann L , Osborne CK , Schiff R . Crosstalk between the estrogen receptor and the HER tyrosine kinase receptor family: molecular mechanism and clinical implications for endocrine therapy resistance. Endocr Rev. 2008;29:217‐233. 18216219 10.1210/er.2006-0045 PMC2528847

[10] Shi JF , Yang N , Ding HJ , et al. ERalpha directly activated the MDR1 transcription to increase paclitaxel‐resistance of ERalpha‐positive breast cancer cells in vitro and in vivo. Int J Biochem Cell Biol. 2014;53:35‐45. 24786296 10.1016/j.biocel.2014.04.016

[11] Sui M , Huang Y , Park BH , Davidson NE , Fan W . Estrogen receptor alpha mediates breast cancer cell resistance to paclitaxel through inhibition of apoptotic cell death. Cancer Res. 2007;67:5337‐5344. 17545614 10.1158/0008-5472.CAN-06-4582

[12] Mason CE , Shu FJ , Wang C , et al. Location analysis for the estrogen receptor‐alpha reveals binding to diverse ERE sequences and widespread binding within repetitive DNA elements. Nucleic Acids Res. 2010;38:2355‐2368. 20047966 10.1093/nar/gkp1188 PMC2853111

[13] Abou‐Kandil A , Eisa N , Jabareen A , Huleihel M . Differential effects of HTLV‐1 Tax oncoprotein on the different estrogen‐induced‐ER alpha‐mediated transcriptional activities. Cell Cycle. 2016;15:2626‐2635. 27420286 10.1080/15384101.2016.1208871 PMC5053584

[14] Wang MM , Traystman RJ , Hurn PD , Liu T . Non‐classical regulation of estrogen receptor‐alpha by ICI182,780. J Steroid Biochem Mol Biol. 2004;92:51‐62. 15544930 10.1016/j.jsbmb.2004.06.002

[15] Alam F , Khan GN , Asad M . *Psoralea corylifolia L*: ethnobotanical, biological, and chemical aspects: a review. Phytother Res. 2018;32(4):597‐615. 29243333 10.1002/ptr.6006 PMC7167735

[16] Limper C , Wang Y , Ruhl S , et al. Compounds isolated from *Psoralea corylifolia* seeds inhibit protein kinase activity and induce apoptotic cell death in mammalian cells. J Pharm Pharmacol. 2013;65:1393‐1408. 23927478 10.1111/jphp.12107

[17] Yin S , Fan CQ , Wang Y , Dong L , Yue JM . Antibacterial prenylflavone derivatives from *Psoralea corylifolia*, and their structure‐activity relationship study. Bioorg Med Chem. 2004;12:4387‐4392. 15265490 10.1016/j.bmc.2004.06.014

[18] Zhao PW , Wang DW , Niu JZ , Wang JF , Wang LQ . Evaluation on phytoestrogen effects of ten kinds of Chinese medicine including flos carthami. Zhongguo Zhong Yao Za Zhi. 2007;32:436‐439. 17511154

[19] Xin D , Wang H , Yang J , et al. Phytoestrogens from *Psoralea corylifolia* reveal estrogen receptor‐subtype selectivity. Phytomedicine. 2010;17:126‐131. 19577453 10.1016/j.phymed.2009.05.015

[20] Ling YH , Liebes L , Zou Y , Perez‐Soler R . Reactive oxygen species generation and mitochondrial dysfunction in the apoptotic response to Bortezomib, a novel proteasome inhibitor, in human H460 non‐small cell lung cancer cells. J Biol Chem. 2003;278:33714‐33723. 12821677 10.1074/jbc.M302559200

[21] Cheng JQ , Lindsley CW , Cheng GZ , Yang H , Nicosia SV . The Akt/PKB pathway: molecular target for cancer drug discovery. Oncogene. 2005;24:7482‐7492. 16288295 10.1038/sj.onc.1209088

[22] Hiscox S , Baruha B , Smith C , et al. Overexpression of CD44 accompanies acquired tamoxifen resistance in MCF7 cells and augments their sensitivity to the stromal factors, heregulin and hyaluronan. BMC Cancer. 2012;12:458. 23039365 10.1186/1471-2407-12-458 PMC3517483

[23] Cho SH , Park YS , Kim HJ , et al. CD44 enhances the epithelial‐mesenchymal transition in association with colon cancer invasion. Int J Oncol. 2012;41:211‐218. 22552741 10.3892/ijo.2012.1453

[24] Kristiansen G , Sammar M , Altevogt P . Tumour biological aspects of CD24, a mucin‐like adhesion molecule. J Mol Histol. 2004;35:255‐262. 15339045 10.1023/b:hijo.0000032357.16261.c5

[25] Lim SC . CD24 and human carcinoma: tumor biological aspects. Biomed Pharmacother. 2005;59(Suppl 2):S351‐S354. 16507407 10.1016/s0753-3322(05)80076-9

[26] Kubatka P , Uramova S , Kello M , et al. Antineoplastic effects of clove buds (*Syzygium aromaticum L*.) in the model of breast carcinoma. J Cell Mol Med. 2017;21:2837‐2851. 28524540 10.1111/jcmm.13197 PMC5661249

[27] Shi JF , Li XJ , Si XX , et al. ERalpha positively regulated DNMT1 expression by binding to the gene promoter region in human breast cancer MCF‐7 cells. Biochem Biophys Res Commun. 2012;427:47‐53. 22975348 10.1016/j.bbrc.2012.08.144

[28] Chen Y , Song J , Jiang Y , Yu C , Ma Z . Predictive value of CD44 and CD24 for prognosis and chemotherapy response in invasive breast ductal carcinoma. Int J Clin Exp Pathol. 2015;8:11287‐11295. 26617852 PMC4637668

[29] Xue P , Yang X , Liu Y , Xiong C , Ruan J . A novel compound RY10‐4 downregulates P‐glycoprotein expression and reverses multidrug‐resistant phenotype in human breast cancer MCF‐7/ADR cells. Biomed Pharmacother. 2014;68:1049‐1056. 25455158 10.1016/j.biopha.2014.10.004

[30] Wu H , Hait WN , Yang JM . Small interfering RNA‐induced suppression of MDR1 (P‐glycoprotein) restores sensitivity to multidrug‐resistant cancer cells. Cancer Res. 2003;63:1515‐1519. 12670898

[31] Adachi Y , Yoshimura M , Nishida K , et al. Acute phase dynamics of circulating tumor cells after paclitaxel and doxorubicin chemotherapy in breast cancer mouse models. Breast Cancer Res Treat. 2018;167:439‐450. 29027049 10.1007/s10549-017-4532-x

[32] Sledge GW Jr , Toi M , Neven P , et al. MONARCH 2: abemaciclib in combination with fulvestrant in women With HR+/HER2‐ advanced breast cancer who had progressed while receiving endocrine therapy. J Clin Oncol. 2017;35:2875‐2884. 28580882 10.1200/JCO.2017.73.7585

[33] Fettig LM , McGinn O , Finlay‐Schultz J , LaBarbera DV , Nordeen SK , Sartorius CA . Cross talk between progesterone receptors and retinoic acid receptors in regulation of cytokeratin 5‐positive breast cancer cells. Oncogene. 2017;36:6074‐6084. 28692043 10.1038/onc.2017.204 PMC5668194

[34] Liu CY , Wu CY , Petrossian K , Huang TT , Tseng LM , Chen S . Treatment for the endocrine resistant breast cancer: current options and future perspectives. J Steroid Biochem Mol Biol. 2017;172:166‐175. 28684381 10.1016/j.jsbmb.2017.07.001

[35] Yue W , Fan P , Wang J , Li Y , Santen RJ . Mechanisms of acquired resistance to endocrine therapy in hormone‐dependent breast cancer cells. J Steroid Biochem Mol Biol. 2007;106:102‐110. 17616457 10.1016/j.jsbmb.2007.05.008 PMC2147683

[36] Clarke R , Skaar TC , Bouker KB , et al. Molecular and pharmacological aspects of antiestrogen resistance. J Steroid Biochem Mol Biol. 2001;76:71‐84. 11384865 10.1016/s0960-0760(00)00193-x

[37] Dal Lago L , D'Hondt V , Awada A . Selected combination therapy with sorafenib: a review of clinical data and perspectives in advanced solid tumors. Oncologist. 2008;13:845‐858. 18695262 10.1634/theoncologist.2007-0233

[38] Verschraegen CF , Skubitz K , Daud A , et al. A phase I and pharmacokinetic study of paclitaxel poliglumex and cisplatin in patients with advanced solid tumors. Cancer Chemother Pharmacol. 2009;63:903‐910. 18682950 10.1007/s00280-008-0813-8

[39] Colleoni M , Viale G , Zahrieh D , et al. Expression of ER, PgR, HER1, HER2, and response: a study of preoperative chemotherapy. Ann Oncol. 2008;19:465‐472. 17986623 10.1093/annonc/mdm509

[40] Jing H , Zhou X , Dong X , et al. Abrogation of Akt signaling by Isobavachalcone contributes to its anti‐proliferative effects towards human cancer cells. Cancer Lett. 2010;294:167‐177. 20167420 10.1016/j.canlet.2010.01.035

[41] Klingbeil P , Natrajan R , Everitt G , et al. CD44 is overexpressed in basal‐like breast cancers but is not a driver of 11p13 amplification. Breast Cancer Res Treat. 2010;120:95‐109. 19350388 10.1007/s10549-009-0380-7

[42] Kim MH , Kim KS , Park MJ , et al. In vivo monitoring of CD44 + cancer stem‐like cells by gamma‐irradiation in breast cancer. Int J Oncol. 2016;48:2277‐2286. 27098303 10.3892/ijo.2016.3493 PMC4864145

